# Probiotic Properties, Prebiotic Fermentability, and GABA-Producing Capacity of Microorganisms Isolated from Mexican Milk Kefir Grains: A Clustering Evaluation for Functional Dairy Food Applications

**DOI:** 10.3390/foods10102275

**Published:** 2021-09-26

**Authors:** Alejandra Hurtado-Romero, Mariano Del Toro-Barbosa, Misael Sebastián Gradilla-Hernández, Luis Eduardo Garcia-Amezquita, Tomás García-Cayuela

**Affiliations:** Tecnologico de Monterrey, Escuela de Ingeniería y Ciencias, Ave. General Ramón Corona 2514, Zapopan 45138, Jalisco, Mexico; a01230908@itesm.mx (A.H.-R.); mtb1508@gmail.com (M.D.T.-B.); msgradilla@tec.mx (M.S.G.-H.); garcia.amezquita@tec.mx (L.E.G.-A.)

**Keywords:** probiotic, prebiotic, psychobiotic, starter culture, dairy, kefir, lactulose, inulin, γ-aminobutyric acid, lactic acid bacteria

## Abstract

Isolation and functional characterization of microorganisms are relevant steps for generating starter cultures with functional properties, and more recently, those related to improving mental health. Milk kefir grains have been recently investigated as a source of health-related strains. This study focused on the evaluation of microorganisms from artisanal Mexican milk kefir grains regarding probiotic properties, in vitro fermentability with commercial prebiotics (lactulose, inulin, and citrus pectin), and γ-aminobutyric acid (GABA)-producing capacity. Microorganisms were identified belonging to genera *Lactococcus*, *Lactobacillus*, *Leuconostoc*, and *Kluyveromyces*. The probiotic properties were assessed by aggregation abilities, antimicrobial activity, antibiotic susceptibility, and resistance to in vitro gastrointestinal digestion, showing a good performance compared with commercial probiotics. Most of isolates maintained a concentration above 6 log colony forming units/mL after the intestinal phase. Specific isolates of *Kluyveromyces* (BIOTEC009 and BIOTEC010), *Leuconostoc* (BIOTEC011 and BIOTEC012), and *Lactobacillus* (BIOTEC014 and BIOTEC15) showed a high fermentability in media supplemented with commercial prebiotics. The capacity to produce GABA was classified as medium for *L. lactis* BIOTEC006, BIOTEC007, and BIOTEC008; *K. lactis* BIOTEC009; *L. pseudomesenteroides* BIOTEC012; and *L. kefiri* BIOTEC014, and comparable to that obtained for commercial probiotics. Finally, a multivariate approach was performed, allowing the grouping of 2–5 clusters of microorganisms that could be further considered new promising cultures for functional dairy food applications.

## 1. Introduction

Functional dairy products account for over 40% of the functional foods market [1]. Most functional dairy products are fermented products such as fermented milk, yogurt, cheese, and yogurt-type products, including low-lactose or lactose-free products. Additionally, these dairy fermented products have long been used as carriers of probiotic microorganisms and prebiotic ingredients [2]. In this context, milk kefir is a fermented dairy product with an increasing popularity due to its nutritional and reported antimicrobial, immunological, antitumor, and hypocholesterolemic effects [3]. It is made from kefir grains, which contain lactic acid bacteria (LAB) and various yeasts combined with caseins and complex sugars in a polysaccharide matrix known as kefiran, which comprises equal amounts of glucose and galactose [3]. The growing popularity of kefir beverages has prompted the use of kefir starters in dairy production. A wide variety of studies emphasize the advantages of milk kefir consumption, showing that the constitution and prevalence of microbial diversity of kefir grains and metabolic fermentation products may differ depending on the carbon and energy sources available for fermentation [4]. The nutritional composition of kefir varies depending on the milk composition, the origin of the grains used, time, the temperature of fermentation, and storage conditions. In addition, milk kefir grains require milk or whey-based medium and sometimes can be grown in plant-based “milk” [5]. As a way of standardizing kefir production, the use of defined cultures has been proposed, being an interesting approach that may eliminate the problems associated with the use of kefir grains. Moreover, using the specific microbiota isolated from kefir grains as a starter culture can produce a fermented food whose properties are close to those of traditional kefir while assuring a high-quality product [6].

Fermentation of food and beverages by probiotic strains have potential health benefits not only by protecting the intestinal barrier, improving nutritional status, or limiting the growth of pathogens but also by influencing brain health with action mechanisms that include the production of neurotransmitters, such as γ-aminobutyric acid (GABA) [7]. GABA is among the primary neurotransmitters of the mammalian central nervous system, whose role is to control excitatory and inhibitory neurotransmission [8]. Several strains of *Bifidobacterium* and *Lactobacillus* have received considerable attention due to their psychobiotic properties, showing mental health benefits [9]. Currently, the food industry is very interested in seeking highly productive GABA strains and optimizing the growth conditions for these bacteria due to the role of this bioactive compound in the treatment of mental disorders as anxiety and depression [10].

Different methodologies have been employed to discover new probiotic strains, being traditional in vitro and in vivo assays along with novel omics the most used approaches. Furthermore, screening for novel probiotic strains includes safety, antimicrobial, and survival assays [11]. In addition, several quantitative techniques are employed regarding the stimulation of probiotics growth, or the activity of microorganisms present in the colon in order to determine the functional activity of prebiotics or non-digestible food ingredients. These methods are based on the measurement of microbial populations, growth rates, substrate assimilation, and/or production of specific metabolites [12].

In recent years, numerous scientific investigations have been published regarding the isolation and characterization of microorganisms from kefir grains of countries like Taiwan, China, Argentina, and Russia. Different species of lactobacilli were identified, such as *Lactobacillus kefiranofaciens*, *Lacticaseibacillus casei*, *Lactiplantibacillus plantarum, Lactobacillus helveticus*, *Lentilactobacillus kefir*, and *Lentilactobacillus parakefir*. Various yeasts were also identified, highlighting *Kluyveromyces marxianus* and *Saccharomyces cerevisiae* [13]. Further, *L. kefiranofaciens* isolated from Taiwan kefir grains showed an anti-colitis effect through in vitro and in vivo tests [14]. On the other hand, yeasts found in Russia and Argentina kefir grains showed tolerance to low pH and bile salts; however, they were not able to adhere to intestinal epithelial cells [15]. Therefore, the isolation, identification, and functional characterization of microorganisms from artisanal milk kefirs are relevant steps for generating starter cultures in the production of novel dairy fermented products with attractive nutritional and functional properties.

The highly variable nature of the microorganisms present in traditional kefir requires a characterization individually in each grain and kefir beverage, especially from Mexico where this research is scarce. Based on that, this study aimed to isolate and identify autochthonous lactic acid bacteria and yeasts from artisanal Mexican milk kefir grains and characterize the probiotic properties, the in vitro fermentability with commercial prebiotics, and the psychobiotic potential through GABA production. Additionally, a multivariate approach with a Cluster Analysis was performed to easily select microbial starters with functional properties to produce fermented dairy products. Commercial probiotic strains *Lactobacillus acidophilus* La3, *Lacticaseibacillus rhamnosus* GG, and *Lactiplantibacillus plantarum* 299v were also included in this study for comparative purposes.

## 2. Materials and Methods

### 2.1. Chemicals, Reagents, Enzymes and Bacterial Strains

All chemicals and reagents used were from analytical grade. Difco MRS (Man-Rogosa-Sharpe, Sparks, MA, USA) media and agar, M17 media and agar, Nutrient Broth, and Potato Dextrose Agar (PDA) were used to isolate and grow microorganisms. Commercial probiotic strains *L. acidophilus* La3, *L. rhamnosus* GG, and *L. plantarum* 299v, and pathogenic-like strains *Escherichia coli* ATCC-25922, *Staphylococcus aureus* ATCC-BAA-42, and *Salmonella typhi* BIOTEC019 were used. Enzymes GABase (G7509), pepsin from porcine gastric mucosa (P7000), α-amylase from porcine pancreas (A3176), pancreatin from porcine pancreas (P1750), and bile salts (B3883) were purchased from Sigma-Aldrich (St. Louis, MO, USA). Gram-positive antibiotic disks Multibac I.D. (Mexico) were used to evaluate antibiotic resistance. To evaluate fermentability with commercial prebiotics, lactulose and agave inulin were purchased from Merck and Enature (México; www.e-nature.com-mx, accessed on 21 September 2021), respectively; dextrose and citric pectin of analytical grade were purchased as well.

### 2.2. Kefir Grains

Kefir grains were obtained from two different artisanal milk kefir beverages in two different locations in Guadalajara, Jalisco State, México. The grains were grown routinely in ultra-pasteurized skim cow milk at room temperature every 24 h, and then they were propagated in the laboratory at the same conditions.

### 2.3. Isolation of Bacteria and Yeast from Kefir Grains

Kefir grains were processed according to the methodology proposed previously [16]. Briefly, 10 g of each source of kefir grains was suspended in 50 mL of sterile saline solution (0.85% NaCl) and homogenized (10–20 min, 5000 rpm) in a T25 homogenizer (IKA Ultra-Turrax). Dilutions were made of each resulting sample and plated in three agars: MRS, M17, and PDA for 48 h at 30 °C or 37 °C and in aerobic and anaerobic conditions. Different colonies were selected, inoculated in the respective media broth (MRS, M17, or Nutrient Broth) at the same conditions, and plated again to isolate a colony from a uniform sample. Moreover, the selected colonies were subjected to gram staining and the catalase test to select presumptive LAB (rods or cocci Gram-positive and catalase-negative). Distinct colonies were isolated and cultured in the respective agar plates at the same conditions for later identification. The final selected isolates were stored at −80 °C.

### 2.4. Microorganism Identification by MALDI-TOF MS

The microorganism identification was performed using Matrix-Assisted Laser Desorption/Ionization Mass Spectrometry (MALDI-TOF MS). For bacteria and yeasts, the biomass of an isolated colony was transferred to a stainless-steel plate following the “Extended Direct Transfer Method” protocol (Bruker Daltonics GmbH, Santa Clara, CA, USA), and mass spectra were generated with the “MBT_FC.par” method in a Microflex LT equipment (Bruker Daltonics GmbH). Using the MALDI BIOTYPER 3.1 software, the spectra obtained were compared with reference spectra from the BDAL database (Bruker Daltonics GmbH). For filamentous fungi, a protein extract was generated from a mycelium pellet of each microorganism following the “Formic Acid Extraction Method” protocol (Bruker Daltonics GmbH). Briefly, 1 μL of this extract was transferred to a stainless-steel plate, allowed to dry and covered with 1 μL of the matrix (10 mg/mL of hydroxycinnamic acid dissolved in acetonitrile:water:trifluoroacetic acid 50:47.5:2.5). Mass spectra were generated with the method “MBT_FC.par” on a Microflex LT equipment (Bruker Daltonics GmbH). Using the MALDI BIOTYPER 3.1 software, the spectra obtained were compared with reference spectra from the FILAMENTOUS FUNGI and BDAL databases (Bruker Daltonics GmbH). The software estimates a score value between 0 and 3 to determine the similarity between the sample and reference spectrum (Table 1). Ten isolates were finally selected from the identified microorganisms for subsequent experiments.

### 2.5. Aggregation Experiments

The first approach for aggregation abilities was a visual screening. Therefore, microorganisms were grown in 2 mL of MRS at 30 °C for 24 h under aerobic conditions. Cultures were vortexed for 15 s, and the auto-aggregation was observed after 3 min under resting conditions (formation of precipitates with a clear observation of supernatants). Additionally, the aggregation phenotype (snowflake aggregates) was monitored after vigorous mixing of the culture. A further spectrophotometric analysis was performed as described by García-Cayuela et al. (2014) [17]. Briefly, bacterial cells (10^8^ CFU/mL) were cultured overnight and harvested by centrifugation (3000× *g*, 20 min, 4 °C), washed twice with phosphate-buffered saline PBS pH 7.1 ± 0.2 and resuspended in the same buffer. Buffer was prepared according to this composition: 10 mM Na_2_HPO_4_, 1 mM KH_2_PO_4_, 140 mM NaCl, 3 mM KCl, pH adjusted with NaOH 0.1 M and HCL 1 M. The mixture was vortexed and incubated at 30 °C for 24 h without agitation, following absorbance values (OD 600) at 0, 2, 6, 20, and 24 h [17]. Aggregation percentage was expressed as follows: $\left[1-\left(\frac{ATime}{A0}\right)\times 100\right)$ where *A*0 represents absorbance at 0 h and *ATime* represents the absorbance of the mixture at different times. Next, co-aggregation assays were done with an overnight culture of isolates or commercial probiotics and pathogen-like strains (*E. coli* ATCC-25922, *S. aureus* ATCC-BAA-42, and *S. typhi* BIOTEC019) following the methodology described above. Then, equal volumes (500 μL) of cells of the isolated microorganisms or control probiotic strains, and pathogen-like strains were mixed in pairs adjusting absorbance (between 0.8 and 1.0) and incubated at 30 °C without agitation, following absorbance values (OD600) at 24 h. Percentage of co-aggregation was calculated as [(Apathog + Aisolate)/2 − (Amix)/(Apathog + Aisolate))/2] × 100, where Apathog and Aisolate) represent the absorbance in control tubes containing only the pathogen or the isolate, respectively, and Amix represents the absorbance of the mix suspension at 24 h.

### 2.6. Antibiotic Susceptibility

All selected microorganisms were cultured overnight at 2% in the proper media. After vortexing, 100 μL of the overnight cultures was distributed uniformly on nutrient agar using sterile L-shaped cell spreaders, and susceptibilities to antibiotics were determined by standard disk-diffusion assays (MultiBac I.D., Mexico DF). Disks contained ampicillin, cephalothin, cefotaxime, dicloxacillin, ciprofloxacin, gentamicin, clindamycin, erythromycin, sulfamethoxazole, penicillin, vancomycin, and tetracycline. Assay consists of deposit on the agar surface previously inoculated with the microorganism, disks impregnated with different antibiotics. The antibiotic diffuses radially, after 18 to 24 h of incubation, the discs appear surrounded by a zone of inhibition. These zones of inhibition were observed after 18 h of incubation at 30 °C andalues were compared with the manufacture’s classification: high resistance (+++), intermediate resistance (++), low resistance/low sensitivity (+), no resistance/sensitivity (−) (MultiBac I.D., Mexico DF), depending on the grade of inhibition (diameter observed around each disk). A plate with inoculum of yeast *K. lactis* BIOTEC009 was used as a control.

### 2.7. Antimicrobial Activity

Screening for antimicrobial activity was performed using the agar diffusion assay. Cell cultures of all microorganisms were grown overnight and centrifuged (10,000× *g*, 10 min). Then, 50 μL of the supernatant was neutralized with NaOH (1 M), previously filtered through a 0.45 μm pore size, and placed in triplicate into wells (5 mm diameter) in nutritive agar plates. No neutralized supernatants were also placed in wells for triplicate following the same methodology. *E. coli* ATCC-25922, *S. aureus* ATCC-BAA-42, and *S. typhi* BIOTEC019 were used to inoculate 5 mL of soft-overlay (0.75% agar) nutrient medium, which was seeded onto the respective agar plates. Zones of growth inhibition were measured after an overnight incubation, and inhibition halos (in millimeters) were reported.

### 2.8. Resistance to Simulated In Vitro Gastrointestinal Digestion

The methodology followed was the improved digestion method (INFOGEST 2.0) based on the standardized protocol developed by the COST INFOGEST network [18] with some modifications. Simulated digestion fluids were composed of KCL (46.7 g/L), KH_2_PO_4_ (68 g/L), NaHCO_3_ (84 g/L), NaCl (120 g/L), and MgCl_2_(H_2_O)_6_ (30 g/L) and were divided into a simulated salivary fluid (SSF), simulated gastric fluid (SGF), and simulated intestinal fluid (SIF). The digestion procedure involved the exposure of microorganisms to three successive digestive phases: oral, gastric, and intestinal. Overnight cell cultures (5 mL) were centrifuged (2000× *g*, 15 min, 4 °C), washed twice, and resuspended in sterile saline solution (0.85% NaCl). After taking an initial sample (1 mL), the oral phase involved a dilution of the culture 1:1 (*v*/*v*) SSF containing amylase (75 mg) at pH 7, and incubated 2 min, 100 rpm, 37 °C; these incubation conditions were constant during the assay. The oral bolus was then diluted 1:1 (*v*/*v*) with SGF and gastric enzymes (pepsin, 160 mg) and incubated at pH 3.0 for 2 h. The gastric chyme was then diluted 1:1 (*v*/*v*) with SIF, bile salts (0.1 mol/L), and pancreatic enzymes (40 mg) and incubated at pH 7 for a further 2 h. CaCl_2_(H_2_O)_2_ (588 g/L) was incorporated along with the enzymes in each phase. The sampling process consisted of taking 1 mL of each culture after each step, then realizing serial dilutions and plating in MRS agar to count viable colonies. Plates were incubated at 30 °C during 48 h, and viable colonies were reported in log colony forming units (CFU)/mL.

### 2.9. Evaluation of Fermentability with Commercial Prebiotics

Microorganisms were cultured overnight at 2% in MRS broth, harvested by centrifugation (3000× *g* for 10 min at 4 °C), washed twice, and resuspended in sterile saline solution (0.85% NaCl). MRS medium (composed of proteose peptone 10 g/L; beef extract 10 g/L; yeast extract 5 g/L; dextrose 20 g/L; polysorbate 80 1 g/L; ammonium citrate 2 g/L; sodium acetate 5 g/L; magnesium sulfate 0.1 g/L; manganese sulfate 0.05 g/L and dipotassium phosphate 2 g/L) was prepared to substitute dextrose with different carbon sources: lactulose, agave inulin, and citric pectin (2%), using glucose as the control. Next, microbial growth was monitored in triplicate in 300 μL wells of sterile 96-well microplates with a lid (Corning, New York, AY, USA). All cultures were grown in aerobic conditions at 30 °C for 48 h. The optical densities at 600 nm of the aerobically-grown cultures were recorded at 60 min intervals with an automated microplate reader (Varioskan Lux, Thermo Fisher Scientific, Waltham, MA, USA). Maximum growth rates and lag parameter (lag) of microorganisms were calculated by fitting the curves to a sigmoid model using the Microsoft Excel add-in DMfit v.2.1 (available at http://www.ifr.ac.uk/safety/DMfit/default.html, accessed on 21 September 2021).

### 2.10. GABA Production

The production of GABA was performed according to the protocol proposed by Tsukatani, Higuchi & Matsumoto (2004) [19], which consists of using the GABase method: an enzymatic mixture of gamma-aminobutyrate glutamate aminotransferase (GABA-T) and succinic semialdehyde dehydrogenase (SSDH), which in the presence of alpha-ketoglutarate and NADP+, produces NADPH which can be quantified spectrophotometrically at 340 nm, and then converted to GABA concentration (mM) using a standard curve. First, microorganisms were incubated for 24 h in MRS broth at 30 °C. Then, the culture was centrifuged, and the supernatant was filtered using a 0.45 µm filter. After, 10 µL of the sample were incubated with the enzymatic mixture for 2 h. Finally, the absorbance was measured using a microplate reader (Varioskan Lux, Thermo Fisher Scientific). This assay was performed with MRS and MRS supplemented with 5 mM monosodium glutamate (MSG), a GABA precursor, and incubated for 48 h [20].

### 2.11. Statistical Analysis

Experiments were performed in triplicate for the determination of all the probiotic properties and fermentation with commercial prebiotics, mean and standard deviation (of three independent measurements) were reported. Analysis of variance (ANOVA) and Tukey tests were used for means comparison using a 0.05 significance level (*p*-value) using the Minitab Software. When only 2 levels were present, the significance of the differences were tested using a paired T-test using the same software.

Cluster analysis was performed to analyze the similarity between the microorganisms, in terms of their probiotic properties, prebiotic fermentability and GABA-production capacity. Cluster analysis is a technique for recognizing and groping similar and near objects within a dataset into clusters based on their characteristics [21]. The Ward’s method was used as the clustering algorithm coupled with the hierarchical method, which is a powerful combination to group cases. The clusters may be clearly identified using the linkage distance Dlink/Dmax, which represents the quotient between the linkage distances for a particular case divided by the maximal linkage distance. The quotient was multiplied by 100 to standardize the linkage distance represented on the *x*-axis [22]. Dendrograms were developed to visually represent the results. Cluster analysis was performed with the software R-3.5.3 using the ggdendro package.

## 3. Results

### 3.1. Microorganism Identification

Twelve microorganisms were identified, as shown in Table 1, of which ten were selected for further assays. Identified microorganisms scores ranged from 1.8 to 2.4, being a reliable identification. Differences in scores can be associated with variations in the database. Most of the microorganisms were LAB belonging to the *Lactobacillus* (3), *Lactoccocus* (4), and *Leuconostoc* (2) genera, while the yeast and fungi identified were *Kluyveromyces* *lactis* (2) and *Penicillium commune* (1), respectively. The study focused on the characterization of bacteria and yeasts; therefore, the isolate *P. commmune* BIOTEC017 was discarded. Likewise, the isolate *Lactoccocus lactis* BIOTEC016 was also discarded due to the similarity of its spectrum with that of *L. lactis* BIOTEC007.

### 3.2. Aggregation Experiments

The results of the visual screening and spectrophotometric aggregation assays are shown in Table 2. An aggregation phenotype was observed in six isolates, rapidly forming aggregates in a stationary phase culture after vortexing (isolates BIOTEC009, BIOTEC010, BIOTEC011, BIOTEC012, BIOTEC013, and BIOTEC014). Besides, in the spectrophotometric aggression assay, a considerable percentage of auto-aggregation (33–94%) was observed in all the isolates, with the highest aggregation percentages at 24 h. An increasing tendency is observed in the aggregation values of all the isolates over time (*p* < 0.05). At 24 h, microorganisms that showed a higher aggregation percentage (>50%) were *L. lactis* BIOTEC007 and BIOTEC008, *L. acidophilus* La3, *L. rhamnosus* GG, *L. kefiri* BIOTEC014, and *L. parakefiri* BIOTEC015. The co-aggregation assay results at 24 h with pathogen-like are shown in Table 3. All microorganisms co-aggregated with pathogens with high percentage values (>55%), ranging from 57 to 98% for *E. coli* ATCC-25922; from 53 to 86% for *S. typhi* BIOTEC019; and from 57 to 88% for *S. aureus* ATCC-BAA-42. High aggregation percentages could be used as a preliminary screening to assess their adhesion properties and potential for pathogen’s competitive exclusion.

### 3.3. Antibiotic Susceptibility

Results of antibiotic susceptibility testing are shown in Table 4. Most of the microorganisms showed a certain level of resistance to each antibiotic. However, *L. lactis* species showed no resistance to most of the antibiotics except gentamicin and erythromycin (BIOTEC007), penicillin, and clindamycin (BIOTEC008). Commercial probiotics and *L. parakefiri* BIOTEC015 showed high resistance to almost all antibiotics except for erythromycin; while *Leuconostoc* species showed high resistance to antibiotics like clindamycin, sulfamethoxazole, and vancomycin. *K. lactis* BIOTEC009, and BIOTEC0010 showed high resistance to all the antibiotics tested. It deserves mentioning that the kit is intended to assess the resistance of Gram (+) bacteria. Other tests aimed to evaluate yeasts could be specifically performed.

### 3.4. Antimicrobial Activity

Results of antimicrobial activity are shown in Table 5. Neutralized and non-neutralized supernatant cultures were used for the test; however, no halos were observed in the neutralized cultures. Therefore, only the halos observed in the non-neutralized cultures were measured (in millimeters) and reported. Halos measured were likely associated with the production of organic acids from the microorganisms. No halos were formed in *Lactoccocus* isolates. The comparison between results of antimicrobial effects is often difficult because of the use of different non-standardized approaches, inoculum preparation techniques, inoculum size, growth medium, incubation conditions, and endpoints determination [23]. In this case, the variability of results depends on both the isolate and the indicator microorganism.

### 3.5. In Vitro Digestion Assay

For the in vitro test of survival to gastrointestinal digestion, samples were taken at each stage of digestion to estimate the survival of each microorganism. These results are shown in Table 6. During the initial phase, all the isolates were at a high concentration level (8–9 log CFU/mL), and after oral digestion some significant differences were observed in the concentration of *L. kefiri* BIOTEC014, while the rest of the isolates remained at high concentrations. After the gastric phase, significant differences were observed in cell concentrations with a 2–3 log reduction in *Lactococcus* species and *L. parakefiri* BIOTEC015. Finally, after the intestinal phase, significant differences were observed between microorganisms, with reduction of 1–4 log in *Lactoccocus* species and 2 log in *K. lactis* and *L. pseudomensenteroides* isolates. For *Lactobacillus* isolates, a reduction from 1.5–2.5 was observed. However, the concentration of commercial probiotics showed a reduction of less than 1 log.

### 3.6. Evaluation of Fermentability with Commercial Prebiotics

**Figure 1:**
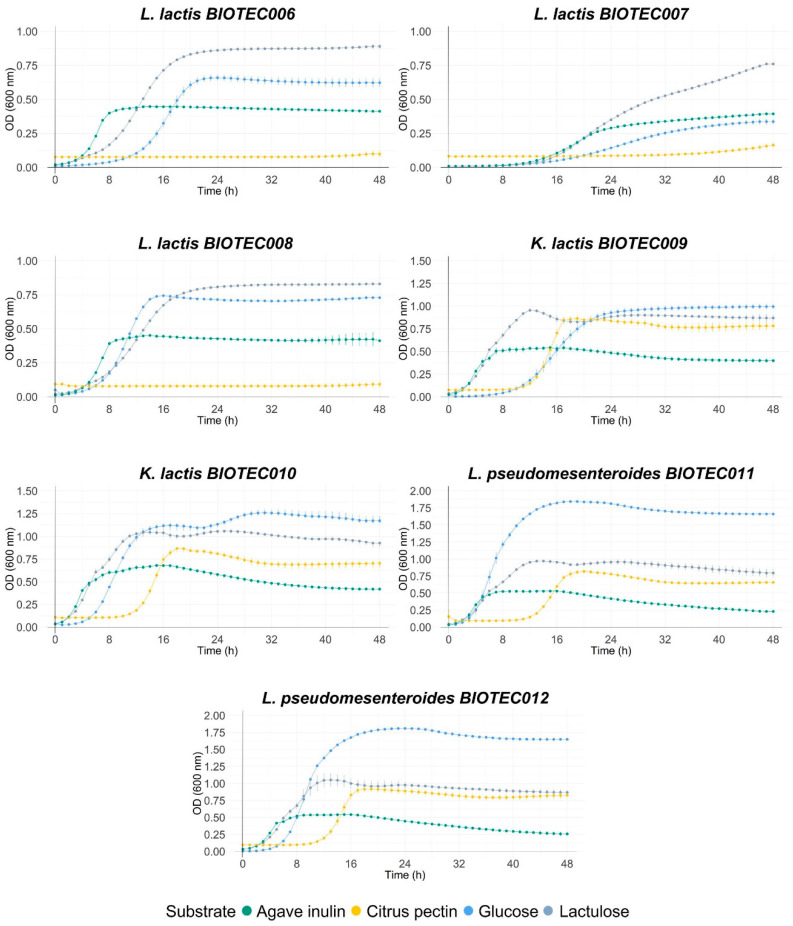

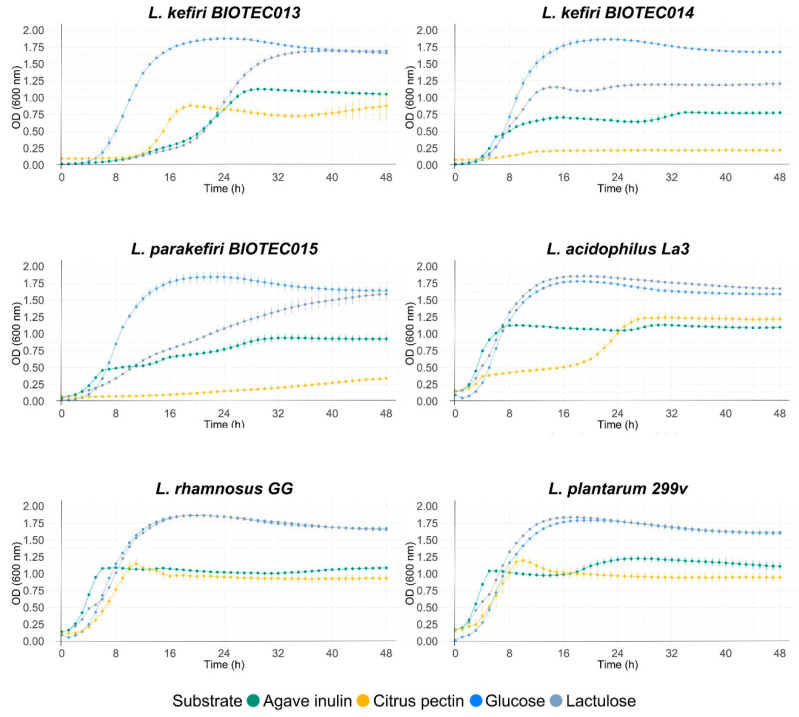
Growth curves of isolated LAB (*Lactoccocus*, *Lactobacillus*, and *Leuconostoc*) and yeast (*Kluyveromyces*) on dextrose, agave inulin, lactulose, and citric pectin (at 1%). Standard deviation was calculated, and curves were done in triplicate.

Different commercial prebiotics were used as a source of fermentable carbon for the isolates of this study. Figure 1 shows the growth curves of microorganisms with the different substrates (lactulose, agave inulin, and citric pectin), using glucose in the control medium. Maximum optical densities at 600 nm (OD_max_), maximum growth rates (µmax), and lag times (h) during the growth of the microorganisms are shown in Table 7. Significant differences were found within all the microorganisms when comparing the growth parameters of each kefir isolate. As expected, most of the isolates grew well on glucose, highlighting the yeast isolates BIOTEC009 and BIOTEC010; and then isolates BIOTEC011, BIOTEC012, BIOTEC13, BIOTEC14, and BIOTEC15. The values for OD_max_ at 600 nm reached between 0.67 and 1.88. These values are related to high growth rates and a shorter latency time (lag).

Commercial probiotics, *K. lactis* BIOTEC009, *K. lactis* BIOTEC010, and *Lactoccocus* isolates grew optimally on lactulose, reaching higher growth rates and OD_max_ values than those reported in glucose media. Agave inulin promoted the growth of commercial probiotics, with OD_max_ values between 1.10 and 1.25, while the growth of the kefir isolates was lower, reaching values between 0.39 and 1.12. Isolates BIOTEC013, BIOTEC14, and BIOTEC15 reached the highest OD_max_ values among this group (0.79–1.12). On the other hand, citrus pectin promoted the growth of only six isolates: *K. lactis* BIOTEC010, *L. pseudomesenteroides* BIOTEC011, *L. kefiri* BIOTEC013, and commercial probiotics. Overall, most microorganisms had similar growth parameters; the maximum growth rate of isolates was constant at high optical density values, demonstrating that they could grow in commercial prebiotics. The main differences between strains were observed for lag times, which varied between 1 and 33 h; the longest lag times were observed for the citrus pectin substrate, while the lag times were shorter for the other three substrates. When evaluating the individual growth of isolates on different prebiotic substrates, statistically significant differences were also found in the growth parameters compared to those reported for the glucose control and within each substrate. Lag times of the microorganisms growing on inulin were shorter than the other substrates, being less than 2 h in most microorganisms. In general, the highest OD_max_ values were recorded for microorganisms growing on glucose and lactulose, highlighting *L. kefiri* BIOTEC014 and *L. parakefiri* BIOTEC015 on glucose, and *L. acidophilus* La3 and *L. rhamnosus* GG on lactulose. However, the highest maximum growth rates were reported for microorganisms growing on lactulose and inulin, highlighting the growth of *L. rhamnosus* GG on lactulose and *L. kefiri* BIOTEC014 on inulin.

### 3.7. GABA Production

**Figure 2:**
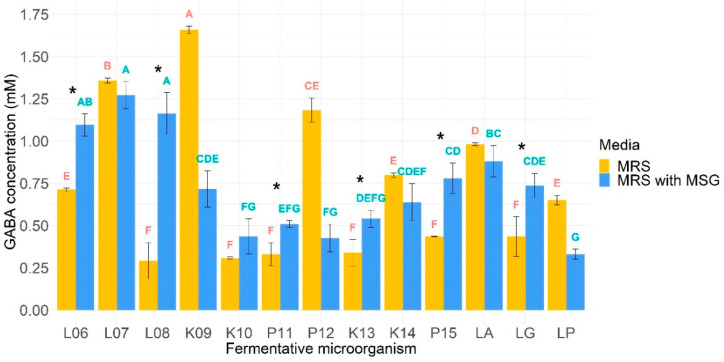
GABA-producing capacity of microorganisms in MRS and MRS supplemented with MSG. Means in the same incubation media that do not share a letter are significantly different. Fermentative microorganisms whose production of GABA increased significantly when incubated with MSG (respect to MRS-with MSG) were denoted with an asterisk. L06, *L. lactis* BIOTEC006; L07, *L. lactis* BIOTEC007; L08, *L. lactis* BIOTEC008; K09, *K. lactis* BIOTEC009; K10, *K. lactis* BIOTEC010; P11, *L. pseudomesenteroides* BIOTEC011; P12, *L. pseudomesenteroides* BIOTEC012; K13, *L. kefiri* BIOTEC013; K14, *L. kefiri* BIOTEC014; P15, *L. parakefiri* BIOTEC015; LA, *L. acidophilus* La3; LG, *L. rhamnosus* GG; LP, L. plantarum 299v.

The ability of the microorganisms to produce GABA was assessed in 2 different conditions: in MRS and MRS supplemented with MSG. The results are shown in Figure 2. As GABA is produced from the decarboxylation of glutamate through glutamate decarboxylase, MRS was supplemented with MSG expecting to increase the production of GABA. In this sense, it was found that the mean production of GABA was significantly higher in MSG supplemented MRS medium only for *L. lactis* BIOTEC006, *L. lactis* BIOTEC008, *L. pseudomesenteroides* BIOTEC011, *L. kefiri* BIOTEC013, *L. parakefiri* BIOTEC015, and *L. rhamnosus* GG. Regarding non-supplemented MRS medium, *K. lactis* BIOTEC009 was the highest GABA producer with 1.66 mM, while the lowest was *L. lactis* BIOTEC008 with 0.29 mM. Furthermore, it can be observed that among the *L. lactis* species, the BIOTEC006 isolate had the greatest GABA production (466% more than the lowest, BIOTEC007), while among the *K. lactis*, the BIOTEC009 isolate had the best performance (539% more than BIOTEC010). For *L. pseudomesenteroides*, BIOTEC012 achieved the highest concentration (359% more than BIOTEC011), just like BIOTEC014 did for *L. kefiri* (236% more than BIOTEC013). On the other hand, the commercial probiotic with the greatest GABA production was *L. acidophilus* La3.

### 3.8. Cluster Analysis

**Figure 3:**
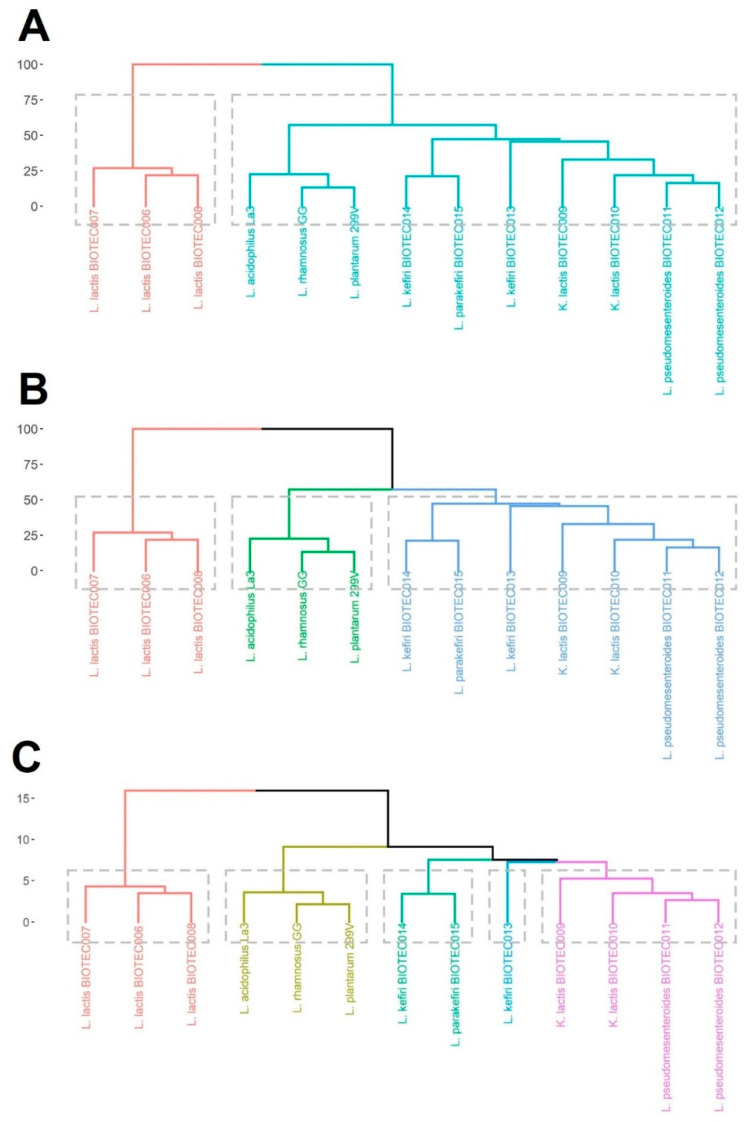
Cluster analysis of the kefir isolates and commercial probiotics. The differences were computed as dissimilarities, and all measured data from the different tests performed were used as input variables. The dashed line was used to represent the cut-off point grouping statistical clusters by using a linkage distance (Dlink/Dmax) × 100 < 60 (**A**), <45 (**B**), and <40 (**C**).

All data from probiotic properties, prebiotic fermentability, and GABA-production capacity were used as input variables to run a cluster analysis and divide the microorganisms in homogeneous groups. Cluster dendrograms are showed in Figure 3. The linkage distance (Dlink/Dmax) × 100 < 60 is a useful criterion to select a number of statistically significant clusters. This defines a partition such that clusters below that height are distant from each other by at least that amount, and the dendrogram suggests the number of clusters. Large changes in fusion levels are taken to indicate the best cut [24]. ((Dlink/Dmax) × 100 < 60 would indicate better cut than (Dlink/Dmax) × 100 < 45 or ((Dlink/Dmax) × 100 < 40). Based on that, there are two main clusters (Figure 3A), one with only the *L. lactis* isolates, and the remaining group is made up the rest of the microorganisms. The *L. lactis* group is the most dissimilar in terms of their overall characteristics. If the inequality (Dlink/Dmax) × 100 < 45 is considered, there would be three clusters (Figure 3B), one grouping the *L. lactis* isolates, a second grouping the commercial probiotics, and a third one grouping the rest of the microorganisms. However, if the inequality (Dlink/Dmax) × 100 < 40 is considered, there would be five clusters (Figure 3C). This multivariate approach is a useful tool to reduce the complexity of the dataset and to select promising candidates within different microbial groups for further validations or applications.

## 4. Discussion

In this study, twelve microorganisms were identified using MALDI-TOF MS, which has been reported as a good alternative to DNA sequencing identification procedures [25]. Recent studies have reported the use of MALDI-TOF MS analysis in fermented foods such as cheese and kimchi, identifying more than eighty LAB species. Further, this analysis has been compared to high-throughput sequencing indicating that similar results were obtained from both methods, enabling accurate identification of microorganisms at the species level as well as analysis of the viable cell communities by only identifying the live microorganisms [26]. The evaluation of functional properties was focused on ten kefir isolates belonging to genera *Lactococcus*, *Lactobacillus*, *Leuconostoc*, and *Kluyveromyces.*

### 4.1. Probiotic Properties

To achieve the desired benefit of probiotic bacteria, isolates tested need to form sufficiently large biomass through aggregation. The capability of bacteria to form cellular aggregates via auto-aggregation or co-aggregation can also contribute to persistence in the intestine [27]. Kefir isolates in the present study showed significant auto-aggregation and co-aggregation with pathogenic-like strains at 24 h. The mechanism of cellular aggregation involves a complex interaction of surface and/or secreted components of the cell. Further, the auto-aggregation ability of cells plays a crucial role in adhesion to intestinal cells and the prevention of pathogen colonization. The highest percentage of auto-aggregation was observed after 24 h, showing a time-dependent increase agreeing with results reported by Krausova et al. (2019) [27]. Co-aggregation is also one of the desired properties for probiotics, and it may play an important role in the gastrointestinal tract by preventing adherence of pathogens to the host tissue [17]. Results presented here can be comparable to those reporting co-aggregation with probiotics and pathogens, in a strain–pathogen combination-dependent manner, where specifically *L. reuteri* VB4 showed a high percentage of co-aggregation (>50%) against pathogen *E. faecalis* ATCC 29212 [28]. Interestingly, most of the isolates showed a higher percentage of co-aggregation than the commercial probiotics. These results suggest the ability of kefir isolates to co-aggregate with pathogens and to compete for adhesion to the epithelial cell surface is a strain-dependent manner.

Regarding the antibiotic susceptibility, most isolates showed certain resistance to the antibiotics tested, as presented in Table 4. Gad et al. (2014) [29] reported high susceptibility of LAB isolates to ampicillin and amoxicillin and more resistance to cephalosporins. Furthermore, a high vancomycin resistance rate was observed. This coincides with the results reported in this assay since *Lactoccocus* isolates did not demonstrate resistance to ampicillin. At the same time, most of the lactobacilli isolated from kefir showed high resistance to vancomycin 29]. Antibiotic resistance of probiotics can be divided into “intrinsic” or “acquired” [30]. Intrinsic or endogenous resistance is inherent to a bacterial species, which may be a desirable characteristic to help restore the host gut microbiota during or after a course of antibiotics with the usage of probiotics. On the other hand, acquired resistance occurs when a bacterium that has been sensitive to antibiotics develops resistance by gene mutation of its DNA or horizontal gene transfer. In this sense, LAB are considered carriers of resistance genes that could propagate their genes within the food chain between food and humans and the environment through these mechanisms [30,31]. Horizontal gene transfer among probiotic strains has been reported for *Lactobacillus gasseri, Lacticaseibacillus paracasei*, *Limosilactobacillus reuteri*, *L. rhamnosus*, *L. plantarum*, and some other probiotics. In this regard, a further analysis is necessary to detect resistant genes in our kefir isolates.

Most of the kefir microorganisms showed antimicrobial activity against tested pathogens, probably due to the production of organic acids and reduction of pH. Traditional fermented products can serve as vehicles for pathogenic bacteria; therefore, antimicrobial activity is an important technological aspect when selecting strains for the controlled production of fermented dairy products. The reduction in pH observed in fermented milk products is associated with lactic acid production and other types of organic acids by fermenting LAB [32]. It has been reported that *L. plantarum* isolated from Xinjiang (a traditional dairy product from China) showed strong antimicrobial activities against *Escherichia coli* and *Salmonella* spp., being organic acids a key role in antimicrobial substances in fermentation broths [33]. Similar results were observed by Arena et al. (2016) [34] when evaluating the antimicrobial effect of *L. plantarum* strains against different pathogenic bacteria, depending mostly on a pH-lowering effect of supernatants and/or on the presence of organic acids [34], which can be compared to the results reported in this study.

As generally recognized, microorganisms to be applied as probiotics should be resistant to gastrointestinal conditions. In this respect, the survival to gastrointestinal digestion depended on the type of microorganism, standing out the high resistance of *Lactobacillus* isolates. The results in this study are consistent with those reporting a high survival rate of *Lactobacillus* spp. isolated from Malaysia kefir [35]. Similar results have been reported in the survival of *Lactobacillus* spp. in fermented milk, which retained a high cell number throughout the digestion and decreased by only 1 log. Gastric stage of digestion reduced cell viability; however, most of isolates remained above 6 log CFU/mL after intestinal phase. Similar results were shown for *L. plantarum* ABHEAU-05 from tepache (a Mexican fermented pineapple drink), concluding that the ability of a microorganism to survive acidic conditions depends directly on the concentration of hydronium ions that accumulate inside the cell [36].

### 4.2. Evaluation of Fermentability with Commercial Prebiotics

Fermentability of prebiotics is an important test to check if these ingredients may be used as additional support for probiotics [37]. This property evaluates the selectively stimulation of microorganisms and determines their activity. Maximum growth rates of kefir microorganisms with lactulose, inulin, and citric pectin can be compared to those reported in the literature. Chatterjee (2016) [38] studied the effect of pectin (0.4%) from different fruit wastes on the growth of LAB, showing for *L. casei* the maximum values with pectin from tomato waste [38]. This is comparable with the data reported in this study as citric pectin promoted the growth of *L. kefiri* BIOTEC013 and commercial probiotics. Additionally, lactulose promoted the growth of lactobacilli isolates *L. kefiri* BIOTEC013 and BIOTEC014, and *L. parakefiri* BIOTEC015, among other isolates. Likewise, Figueroa-González (2019) [39] indicated lactulose generates good growth of several strains of lactobacilli (*L. rhamnosus* and *L. casei*). It has been reported that yogurts or fermented milk supplemented with lactulose (4%) may enhance the acidification rate of these products and promote the growth of co-cultures of *L. acidophilus*, *L. rhamnosus*, and *Bifidobacterium lactis* in combination with *Streptococcus thermophilus* [40]. However, it is also important to consider the metabolic system of each strain due to the variations found in the utilization of carbohydrates as a carbon source [39].

In our study, agave inulin promoted the growth of all microorganisms, except for *L. lactis* isolates. Agave inulin has also been reported to favor the growth of probiotic bacteria such as *Ligilactobacillus salivarius* and *Enterococcus faecium*. The growth was related to the molecular structure of the polymer, composed of linear fructose chains. In addition, the degree of polymerization of the molecule affects the degradation of inulin, promoting greater solubility, which favors its degradation and use [41]. Another *Kluyveromyces* species has been reported to ferment a fructan similar to inulin (agavin); *Kluyveromyces marxianus*, isolated from residua of the tequila industry, produces a dimeric β-D-fructan fructohydrolase, with exo-inulinase activity on agavin and inulin [42]. Similarly, Garcia-Gamboa et. al. (2018) [43] reported that probiotics *L. casei* and *L. paracasei* can metabolize agave fructans obtained from several species (*Agave salmiana*, *Agave atrovirens*, and *Agave tequilana*) [43].

### 4.3. Psychobiotic Potential through GABA Production Assay

The psychobiotic potential of the fermentative microorganisms was assessed by studying their ability to secrete GABA, a neuroactive molecule. According to Del Toro-Barbosa et al. (2020) [9], analyzing the production of this neurotransmitter is one of the first in vitro approaches that can be carried out to screen the psychobiotic potential of microorganisms. Tsukatani, Higuchi, and Matsumoto (2004) [19] evaluated the ability of 381 strains of LAB to produce GABA in MRS medium. They classified them according to the amount of GABA produced as low (less than 0.5 mM), medium (from 0.5 to 2.1 mM), and high (more than 2.1 mM). With that proposed classification system, kefir isolates BIOTEC006, BIOTEC007, BIOTEC009, BIOTEC012, BIOTEC014; and commercial probiotics La3, and 299v were considered medium-level GABA-producers, and the rest as low-producing microorganisms when cultured in MRS medium. Redruello et al. (2021) [44] investigated GABA production in six *L. lactis* strains isolated from camel milk. To evaluate their production, they were grown in M17 broth supplemented with glucose for 5 days with GMS 5 mM. Their results showed that all the strains produced an amount of GABA that ranged from 1.22 to 1.80 mM, similar to the values found for the three kefir-isolated strains when incubated with GMS [44]. In another study, Bhanwar et al. (2013) [45] worked with a *L. lactis* strain isolated from yam pickles. They found that when incubated at 30 °C for 48 h and MRS supplemented with MSG at 5%, concentrations of up to 10.7 mM could be achieved [45]. Among the studied commercial probiotics, *L. acidophilus* La3 had the best performance of 0.98 mM, suggesting that the production of this molecule could be highly dependent on the strain and culture medium.

On the other hand, Perpetuini et al. (2020) investigated the production of GABA of 10 different *Kluyveromyces marxianus* strains. The production of GABA for the strains in yeast extract peptone dextrose medium with MSG 10 mm incubated at 27 °C, varied from 2.54 mg/L to 7.78 mg/L (0.025 and 0.075 mM, respectively), which is much lower than the levels found in the two *K. lactis* isolates in this study: 1.65 mM for BIOTEC009 and 0.31 mM for BIOTEC010. In this respect, polymorphisms in the genes coding for glutamate decarboxylase are responsible for variations in GABA production of strains even between the same species [46]. Finally, note that *L. pseudomesenteroides* BIOTEC012 was one of the microorganisms that produced the most GABA content (1.18 mM). In a study carried out by Demribas et al. (2017) [47], a *L. pseudomesenteroides* strain (N-13) isolated from sourdough was able to produce 10.02 mM of GABA when grown in MRS containing 53 mM of MSG for 96 h at 30 °C. All of this suggests that the incubation most likely affects the production of GABA. Evaluation of other products generated in the fermentation can be interesting. Although, in this study, a preliminary evaluation of substrate use was performed with commercial prebiotics. Currently, microorganisms that present the best results in preliminary studies are being evaluated with a series of novel prebiotic substrates with the purpose to evaluate the production of some other interesting bioactive molecules such as lactic acid or short-chain fatty acids.

### 4.4. Choice of Suitable Microorganisms for Potential Functional Dairy Food Applications

One of the main difficulties when selecting microorganisms with specific properties is handling a large amount of data derived from different tests. A multivariable technique based on a cluster analysis was performed as a screening tool to select the best candidates. All data from the assayed kefir isolates and commercial probiotics were submitted to the statistical analysis allowing the grouping of two, three, or five clusters, depending on the Dlink/Dmax calculation [22]. In either case, a clear differentiation is observed between *L. lactis* isolates, commercial probiotics, and other microorganisms. The *L. lactis* isolates stood out for good aggregations properties and GABA-production capacity, acceptable resistance to gastrointestinal conditions, and moderate ability to use prebiotics. Multiple strains of *L. lactis* have been considered as excellent starter cultures [48]. Thus, the BIOTEC006, BIOTEC007, and BIOTEC008 isolates could be used as starter cultures with multiple functional aptitudes for dairy fermented products. For the rest of kefir microorganisms, the isolates of *Kluyveromyces*, *Lactobacillus*, and *Leuconostoc* would be more linked to be used in more specific dairy applications that involve the prebiotic or psychobiotic capacity. Nevertheless, further experiments are required to assess the behavior of the selected isolates to establish the technological and therapeutic benefits [49].

## 5. Conclusions

These results suggest that selected Mexican artisanal milk kefir isolates reported in this study can be successfully used in the design of new dairy fermented products. Additionally, they could be good candidates for further deeper studies regarding probiotic, prebiotic, and psychobiotic characteristics. In fact, we are performing new studies that will elucidate the functional potential of these newly isolated microorganisms.

## Figures and Tables

**Table 1:** MALDITOF-MS identification of kefir microorganisms with software reliability scores ^a^.

MALDI-TOF Result	Assigned Code	MALDI-TOF Score	Reliability
*Lactococcus lactis*	BIOTEC006	2.143	Genus-level, probable species
*Lactococcus lactis*	BIOTEC007	2.446	Genus-level, species level
*Lactococcus lactis*	BIOTEC008	2.400	Genus-level, species level
*Kluyveromyces lactis*	BIOTEC009	2.102	Genus-level, probable species
*Kluyveromyces lactis*	BIOTEC010	1.937	Probable genus
*Leuconostoc pseudomesenteroides*	BIOTEC011	1.821	Probable genus
*Leuconostoc pseudomesenteroides*	BIOTEC012	1.885	Probable genus
*Lentilactobacillus* *kefiri*	BIOTEC013	2.006	Genus-level, probable species
*Lentilactobacillus kefiri*	BIOTEC014	2.226	Genus-level, probable species
*Lentilactobacillus parakefiri*	BIOTEC015	2.151	Genus-level, probable species
*Lactococcus lactis*	BIOTEC016	1.861	Probable genus
*Penicillium commune*	BIOTEC017	2.366	Genus-level, species level

^a^ Reliability score: 2.300 to 3.000 correspond to high reliability at the species level, 2.000 to 2.299 high reliability at the genus level and probable species identification, 1.700 to 1.999 probable identification at the genus level and <1.699 unreliable identification.

**Table 2:** Auto-aggregation abilities of the kefir isolates and commercial probiotics ^a^.

Microorganisms ^b^	Auto-Aggregation (%)			
	2 h	6 h	20 h	24 h
*L. acidophilus* La3 (−)	4.36 ± 0.29 ^cd^	10.29 ± 0.10 ^de^	56.54 ± 0.12 ^c^	72.21 ± 0.53 ^c^
*L. rhamnosus* GG (−)	3.26 ± 0.29 ^f^	8.17 ± 0.19 ^ef^	35.17 ± 1.76 ^e^	53.05 ± 0.62 ^ef^
*L. plantarum* 299V (−)	3.54 ± 0.35 ^def^	8.00 ± 0.21 ^f^	35.86 ± 3.51 ^e^	46.98 ± 3.12 ^fg^
*L. lactis* BIOTEC006 (−)	2.97 ± 0.03 ^f^	12.48 ± 0.44 ^c^	30.85 ± 1.06 ^ef^	38.87 ± 2.52 ^gh^
*L. lactis* BIOTEC007 (−)	10.64 ± 0.15 ^a^	31.78 ± 1.08 ^b^	77.00 ± 1.94 ^b^	84.31 ± 1.28 ^b^
*L. lactis* BIOTEC008 (−)	10.09 ± 0.00 ^a^	32.24 ± 0.95 ^b^	85.93 ± 0.85 ^a^	94.33 ± 0.52 ^a^
*K. lactis* BIOTEC009 (+)	4.14 ± 0.30 ^de^	9.50 ± 0.54 ^def^	28.46 ± 0.41 ^f^	46.01 ± 0.38 ^h^
*K. lactis* BIOTEC010 (+)	5.12 ± 0.19 ^bc^	10.80 ± 0.38 ^cd^	28.82 ± 0.53 ^f^	39.00 ± 3.77 ^gh^
*L. pseudomesenteroides* BIOTEC011 (+)	3.43 ± 0.29 ^ef^	7.48 ± 0.46 ^f^	26.00 ± 0.20 ^f^	33.99 ± 1.42 ^h^
*L. pseudomesenteroides* BIOTEC012 (+)	3.50 ± 0.12 ^ef^	9.27 ± 0.13 ^def^	26.96 ± 0.28 ^f^	33.92 ± 0.29 ^h^
*L. kefiri* BIOTEC013 (+)	5.33 ± 0.17 ^b^	9.05 ± 0.43 ^def^	27.67 ± 0.29 ^f^	32.60 ± 3.12 ^h^
*L. kefiri* BIOTEC014 (+)	10.53 ± 0.07 ^a^	34.73 ± 0.42 ^a^	57.81 ± 0.04 ^c^	59.87 ± 0.03 ^de^
*L. parakefiri* BIOTEC015 (−)	2.80 ± 0.01 ^f^	10.44 ± 0.68 ^cd^	50.14 ± 2.73 ^d^	66.10 ± 0.65 ^cd^

^a^ Data are expressed as % of auto-aggregation measured after 2, 6, 20, and 24 h of incubation. The values are means of duplicate measurements ± standard deviation. Different letters in the same column denote significant differences among all the microorganisms studied. ^b^ Aggregation phenotype is indicated as positive (+) or negative (−).

**Table 3:** Co-aggregation abilities of isolates and controls with *E. coli* ATCC-25922, *S. typhi* BIOTEC019, and *S. aureus* ATCC-BAA-42 at 24 h ^a^.

Microorganisms	Co-Aggregation with Pathogens (%)		
	*E. coli* ATCC-25922	*S. typhi* BIOTEC019	*S. aureus* ATCC-BAA-42
*L. acidophilus* La3	68.90 ± 1.15 ^c^	63.82 ± 0.07 ^e^	73.84 ± 1.35 ^efg^
*L. rhamnosus* GG	58.05 ± 0.38 ^de^	63.48 ± 1.13 ^ef^	70.83 ± 0.02 ^fg^
*L. plantarum* 299V	62.26 ± 0.86 ^cde^	53.76 ± 0.22 ^g^	76.55 ± 0.72 ^def^
*L. lactis* BIOTEC006	83.99 ± 1.77 ^b^	86.41 ± 1.09 ^a^	84.35 ± 0.15 ^cd^
*L. lactis* BIOTEC007	85.57 ± 0.81 ^b^	77.74 ± 0.62 ^bc^	86.01 ± 0.67 ^de^
*L. lactis* BIOTEC008	85.24 ± 0.74 ^b^	82.55 ± 1.92 ^ab^	83.49 ± 0.50 ^bcd^
*K. lactis* BIOTEC009	83.64 ± 0.46 ^b^	63.57 ± 0.11 ^ef^	83.18 ± 0.94 ^abc^
*K. lactis* BIOTEC010	62.94 ± 1.30 ^cde^	58.60 ± 0.15 ^fg^	57.63 ± 0.42 ^h^
*L. pseudomesenteroides* BIOTEC011	68.58 ± 1.73 ^cd^	62.58 ± 0.19 ^ef^	71.27 ± 0.01 ^efg^
*L. pseudomesenteroides* BIOTEC012	63.22 ± 0.48 ^e^	75.62 ± 0.33 ^cd^	72.45 ± 0.53 ^g^
*L. kefiri* BIOTEC013	57.01 ± 0.57 ^e^	70.64 ± 2.41 ^d^	72.87 ± 0.57 ^fg^
*L. kefiri* BIOTEC014	98.13 ± 0.29 ^a^	73.12 ± 2.92 ^cd^	88.38 ± 0.61 ^a^
*L. parakefiri* BIOTEC015	98.76 ± 0.22 ^a^	84.70 ± 1.03 ^a^	79.79 ± 0.19 ^ab^

^a^ Data are expressed as % of co-aggregation measured after 24 h of incubation. The values are means of duplicate measurements ± standard deviation. Different letters in the same column denote significant differences among all the microorganisms studied.

**Table 4:** Antibiotic resistance of the identified kefir isolates and commercial probiotics.

Microorganisms	Antibiotics											
	AM	CF	CFX	DC	CPF	GE	CLM	E	STX	PE	VA	TE
*L. acidophilus* La3	+	+ +	+ + +	+ + +	+ + +	+ + +	−	−	+	+ + +	+ + +	+
*L. rhamnosus* GG	+ + +	+ + +	+ + +	+ + +	+ +	+ + +	+	−	+ + +	+ +	+ + +	+ + +
*L. plantarum* 299V	+ +	+ + +	+ + +	+ + +	+ +	+ + +	+	−	+ + +	+ +	+ + +	+ +
*L. lactis* BIOTEC006	−	−	−	−	−	−	−	−	−	−	−	−
*L. lactis* BIOTEC007	−	−	−	−	−	+ + +	−	+ + +	−	−	−	−
*L. lactis* BIOTEC008	−	−	−	+ +	+ +	+ +	+ + +	−	−	+ + +	+	−
*K. lactis* BIOTEC009	+ + +	+ + +	+ + +	+ + +	+ + +	+ + +	+ + +	+ + +	+ + +	+ + +	+ + +	+ + +
*K. lactis* BIOTEC010	+ + +	+ + +	+ + +	+ + +	+ + +	+ + +	+ + +	+ + +	+ + +	+ + +	+ + +	+ + +
*L. pseudomesenteroides* BIOTEC011	+ +	−	−	+ +	−	−	+ + +	−	+ +	−	+ + +	−
*L. pseudomesenteroides* BIOTEC012	+ +	−	−	+ +	−	−	+ + +	−	+ + +	−	+ +	−
*L. kefiri* BIOTEC013	+	−	+	+ + +	−	−	+	−	+ + +	+	−	−
*L. kefiri* BIOTEC014	−	−	−	−	+ + +	−	−	−	−	+	+ +	+ + +
*L. parakefiri* BIOTEC015	+ + +	+ + +	+ +	+ + +	+ +	+ + +	+	−	+ + +	+ + +	+ + +	+

+ + +, High resistance; + + Intermediate resistance; + Low resistance; −, No resistance. AM, Ampicilin; CF, Cephalothin; CFX, Cefotaxime; DC, Dicloxacilin; CPF, Ciprofloxacin; GE, Gentamicin; CLM, Clindamycin; E, Erythromycin; STX, Su famethoxazole; PE, Penicilin; VA, Vancomycin; TE, Tetracycline.

**Table 5:** Evaluation of the antimicrobial activity of kefir isolates measured in non-neutralized cultures against pathogens ^a^.

Microorganisms	Media Growth Inhibition Halos (in mm)		
	*E. coli* ATCC-25922	*S. typhi* BIOTEC019	*S. aureus* ATCC-BAA-42
*L. acidophilus* La3	18.33 ± 1.53 ^a^	16.67 ± 0.58 ^ab^	14.67 ± 0.58 ^b^
*L. rhamnosus* GG	18.67 ± 1.53 ^a^	17.67 ± 1.53 ^a^	14.67 ± 0.58 ^a^
*L. plantarum* 299V	19.00 ± 1.00 ^a^	18.33 ± 0.58 ^a^	15.33 ± 0.58 ^b^
*L. lactis* BIOTEC006	ND	ND	ND
*L. lactis* BIOTEC007	ND	ND	ND
*L. lactis* BIOTEC008	ND	ND	ND
*K. lactis* BIOTEC009	14.67 ± 2.08 ^a^	12.00 ± 1.00 ^a^	15.33 ± 0.58 ^a^
*K. lactis* BIOTEC010	14.33 ± 1.15 ^a^	12.00 ± 0.00 ^b^	15.33 ± 0.58 ^a^
*L. pseudomesenteroides* BIOTEC011	13.00± 1.00 ^b^	12.00 ± 0.00 ^b^	15.33 ± 0.58 ^a^
*L. pseudomesenteroides* BIOTEC012	15.00 ± 1.00 ^a^	13.00 ± 0.00 ^b^	16.33 ± 0.58 ^a^
*L. kefiri* BIOTEC013	17.00 ± 1.15 ^a^	14.67 ± 0.58 ^b^	16.00 ± 1.00 ^ab^
*L. kefiri* BIOTEC014	16.33 ± 1.15 ^a^	14.67 ± 0.58 ^a^	16.33 ± 0.58 ^a^
*L. parakefiri* BIOTEC015	16.00 ± 0.58 ^ab^	14.33 ± 0.58 ^b^	15.33 ± 0.58 ^a^

^a^ The values measured are means of triplicate measurements ± standard deviation. Different letters in each row denote significant differences among the production of organic acid against pathogens. ND indicates values not determined.

**Table 6:** Survival of individual kefir isolates and commercial probiotics to in vitro gastrointestinal digestion simulation ^a^.

Microorganisms	LOG (CFU/mL)			
	Initial Phase	Oral Phase	Gastric Phase	Intestinal Phase
*L. acidophilus* La3	9.41 ± 0.01 ^b^	9.62 ± 0.01 ^a^	8.88 ± 0.02 ^c^	9.15 ± 0.01 ^d^
*L. rhamnosus* GG	9.59 ± 0.16 ^a^	9.54 ± 0.09 ^a^	8.56 ± 0.03 ^b^	8.77 ± 0.08 ^b^
*L. plantarum* 299V	9.55 ± 0.07 ^b^	9.79 ± 0.02 ^a^	8.60 ± 0.02 ^d^	8.85 ± 0.02 ^c^
*L. lactis* BIOTEC006	8.39 ± 0.12 ^a^	8.38 ± 0.12 ^a^	4.84 ± 0.08 ^b^	4.75 ± 0.21 ^b^
*L. lactis* BIOTEC007	8.00 ± 0.00 ^b^	8.30 ± 0.00 ^a^	6.95 ± 0.07 ^c^	6.86 ± 0.05 ^c^
*L. lactis* BIOTEC008	9.35 ± 0.49 ^a^	8.65 ± 0.07 ^a^	5.45 ± 0.21 ^b^	5.22 ± 0.21 ^b^
*K. lactis* BIOTEC009	8.74 ± 0.06 ^a^	8.94 ± 0.14 ^a^	6.75 ± 0.21 ^b^	6.60 ± 0.00 ^b^
*K. lactis* BIOTEC010	8.96 ± 0.05 ^a^	9.09 ± 0.02 ^a^	6.99 ± 0.12 ^b^	6.75 ± 0.21 ^b^
*L. pseudomesenteroides* BIOTEC011	9.24 ± 0.09 ^a^	9.05 ± 0.08 ^a^	6.78 ± 0.17 ^b^	6.95 ± 0.04 ^b^
*L. pseudomesenteroides* BIOTEC012	8.80 ± 0.02 ^a^	9.02 ± 0.03 ^a^	6.90 ± 0.00 ^b^	6.99 ± 0.12 ^b^
*L. kefiri* BIOTEC013	9.09 ± 0.12 ^a^	9.15 ± 0.21 ^a^	6.58 ± 0.00 ^b^	6.56 ± 0.17 ^b^
*L. kefiri* BIOTEC014	8.09 ± 0.04 ^a^	7.84 ± 0.00 ^b^	6.46 ± 0.06 ^c^	6.50 ± 0.00 ^c^
*L. parakefiri* BIOTEC015	8.09 ± 0.05 ^a^	7.54 ± 0.01 ^a^	5.86 ± 0.07 ^b^	5.60 ± 0.01 ^c^

^a^ The values measured are means of triplicate measurements ± standard deviation. Different letters in each column denote significant differences among each individual strain and the phases of the digestion.

**Table 7:** Maximum optical density at 600 nm (ODmax), maximum growth rate (μmax, h^−1^), and lag (h) parameters of bacteria growing under aerobic conditions on glucose, lactulose, agave inulin, and citric pectin as carbon sources.

Microorganisms		Glucose	Lactulose	Agave Inulin	Citric Pectin
*L. acidophilus* La3	OD_max_	1.78 ± 0.01 ^Bb^	1.87 ± 0.00 ^Aa^	1.14 ± 0.00 ^Db^	1.27± 0.04 ^Cb^
	μ_max_	0.21 ± 0.00 ^Bcd^	0.19 ± 0.00 ^Ca^	0.22 ± 0.00 ^Aa^	0.09 ± 0.00 ^De^
	lag	1.68 ± 0.10 ^Cij^	2.04 ± 0.02 ^Bg^	1.01 ± 0.03 ^Df^	16.76 ± 0.07 ^Ac^
*L. rhamnosus* GG	OD_max_	1.87 ± 0.02 ^Aa^	1.87 ± 0.01 ^Aa^	1.10 ± 0.01 ^Cc^	1.38 ± 0.02 ^Ba^
	μ_max_	0.21 ± 0.00 ^Bbcd^	0.18 ± 0.00 ^Cb^	0.25 ± 0.00 ^Aa^	0.17 ± 0.00 ^Dc^
	lag	3.01 ± 0.09 ^Ah^	3.22 ± 0.01 ^Af^	1.25 ± 0.04 ^Cef^	2.46 ± 0.39 ^Bf^
*L. plantarum* 299v	OD_max_	1.81 ± 0.00 ^Ab^	1.84 ± 0.01 ^Aa^	1.25 ± 0.01 ^Ba^	1.26 ± 0.03 ^Bb^
	μ_max_	0.19 ± 0.00 ^Ae^	0.19 ± 0.00 ^Aab^	0.23 ± 0.05 ^Aa^	0.18 ± 0.00 ^Ab^
	lag	1.26 ± 0.08 ^Aj^	2.03 ± 0.03 ^Ag^	1.55 ± 0.01 ^Ae^	1.80 ± 0.63 ^Af^
*L. lactis* BIOTEC006	OD_max_	0.67 ± 0.03 ^Bf^	0.89 ± 0.01 ^Agh^	0.45 ± 0.00 ^Ch^	0.11 ± 0.02 ^Dh^
	μ_max_	0.08 ± 0.03 ^Bh^	0.07 ± 0.01 ^Cf^	0.10 ± 0.00 ^Abc^	0.00 ± 0.02 ^Dg^
	lag	11.87 ± 0.03 ^Bb^	6.64 ± 0.01 ^Cc^	3.87 ± 0.00 ^Cc^	38.90 ± 0.02 ^Aa^
*L. lactis* BIOTEC007	OD_max_	0.35 ± 0.01 ^Cg^	0.76 ± 0.01 ^Ai^	0.39 ± 0.01 ^Bi^	0.15 ± 0.00 ^Dh^
	μ_max_	0.01 ± 0.00 ^Ci^	0.03 ± 0.00 ^Ah^	0.02 ± 0.00 ^Bd^	0.00 ± 0.00 ^Dg^
	lag	13.10 ± 0.11 ^Da^	11.02 ± 0.07 ^Ab^	10.74 ± 0.02 ^Bb^	32.04 ± 0.71 ^Cb^
*L. lactis* BIOTEC008	OD_max_	0.74 ± 0.00 ^Be^	0.83 ± 0.00 ^Ahi^	0.46 ± 0.00 ^Ch^	0.10 ± 0.01 ^Dh^
	μ_max_	0.11 ± 0.00 ^Ag^	0.06 ± 0.00 ^Cf^	0.09 ± 0.00 ^Bc^	0.00 ± 0.00 ^Dg^
	lag	7.03 ± 0.05 ^Bd^	5.76 ± 0.02 ^Cd^	3.20 ± 0.01 ^Dd^	39.57 ± 0.78 ^Aa^
*K. lactis* BIOTEC009	OD_max_	1.01 ± 0.01 ^Ad^	0.96 ± 0.02 ^Bfg^	0.55 ± 0.01 ^Dg^	0.88 ± 0.02 ^Cde^
	μ_max_	0.08 ± 0.00 ^Ch^	0.12 ± 0.00 ^Bde^	0.11 ± 0.01 ^Bbv^	0.19 ± 0.00 ^Aa^
	lag	9.57 ± 0.30 ^Bc^	1.37 ± 0.15 ^Ci^	1.16 ± 0.08 ^Cf^	12.59 ± 0.16 ^Ade^
*K. lactis* BIOTEC010	OD_max_	1.28 ± 0.01 ^Ac^	1.07 ± 0.00 ^Bd^	0.69 ± 0.00 ^Df^	0.87 ± 0.01 ^Cde^
	μ_max_	0.14 ± 0.01 ^Bf^	0.12 ± 0.00 ^Cd^	0.10 ± 0.00 ^Dbc^	0.17 ± 0.00 ^Abc^
	lag	5.02 ± 0.25 ^Bf^	1.59 ± 0.08 ^Chi^	0.00 ± 0.00 ^Dg^	12.23 ± 0.10 ^Ae^
*L. pseudomesenteroides* BIOTEC011	OD_max_	1.86 ± 0.00 ^Aa^	0.99 ± 0.01 ^Bef^	0.53 ± 0.00 ^Dg^	0.82 ± 0.02 ^Ce^
	μ_max_	0.22 ± 0.00 ^Abcd^	0.11 ± 0.00 ^De^	0.13 ± 0.00 ^Cb^	0.16 ± 0.00 ^Bd^
	lag	1.99 ± 0.02 ^Bi^	1.81 ± 0.05 ^Cgh^	1.29 ± 0.01 ^Def^	12.78 ± 0.10 ^Ade^
*L. pseudomesenteroides* BIOTEC012	OD_max_	1.81 ± 0.00 ^Ab^	1.03 ± 0.08 ^Bde^	0.54 ± 0.01 ^Cg^	0.94 ± 0.02 ^Bc^
	μ_max_	0.22 ± 0.00 ^Abc^	0.12 ± 0.02 ^Cd^	0.11 ± 0.00 ^Cbc^	0.19 ± 0.01 ^Ba^
	lag	5.60 ± 0.05 ^Be^	1.55 ± 0.29 ^Chi^	1.30 ± 0.01 ^Cef^	12.08 ± 0.21 ^Ae^
*L. kefiri* BIOTEC013	OD_max_	1.87 ± 0.02 ^Aa^	1.70 ± 0.01 ^Bb^	1.12 ± 0.01 ^Cbc^	0.90 ± 0.02 ^Dcd^
	μ_max_	0.21 ± 0.01 ^Ade^	0.13 ± 0.00 ^Cd^	0.08 ± 0.00 ^Dc^	0.16 ± 0.00 ^Bd^
	lag	5.50 ± 0.32 ^De^	17.27 ± 0.15 ^Aa^	14.81 ± 0.40 ^Ba^	12.17 ± 0.12 ^Ce^
*L. kefiri* BIOTEC014	OD_max_	1.88 ± 0.01 ^Aa^	1.23 ± 0.02 ^Bc^	0.79 ± 0.00 ^Ce^	0.23 ± 0.00 ^Dg^
	μ_max_	0.24 ± 0.00 ^Aa^	0.14 ± 0.00 ^Bc^	0.08 ± 0.01 ^Cc^	0.02 ± 0.00 ^Df^
	lag	4.96 ± 0.00 ^Af^	4.29 ± 0.06 ^Be^	1.54 ± 0.00 ^Ce^	4.13 ± 0.31 ^Bf^
*L. parakefiri* BIOTEC015	OD_max_	1.88 ± 0.04 ^Aa^	1.64 ± 0.02 ^Bb^	0.95 ± 0.04 ^Cd^	0.35 ± 0.01 ^Df^
	μ_max_	0.22 ± 0.00 ^Ab^	0.04 ± 0.00 ^Bg^	0.03 ± 0.00 ^Cd^	0.01 ± 0.00 ^Dg^
	lag	4.42 ± 0.11 ^Bg^	0.00 ± 0.00 ^Cj^	0.00 ± 0.00 ^Cg^	14.84 ± 1.23 ^Acd^

The values measured are means of triplicate measurements ± standard deviation. Different capital letters in each column indicate significant differences between microorganisms within each substrate. Different lower-case letters in each line indicate significant differences between substrates within each microorganism.

## Data Availability

Not applicable.

## References

[1] Ortiz Y., García-Amézquita E., Acosta C.H., Sepúlveda D.R., Barbosa-Cánovas G.V., María Pastore G., Candoğan K., Medina Meza I.G., Caetano da Silva Lannes S., Buckle K., Yada R.Y., Rosenthal A. (2017). Functional Dairy Products. Global Food Security and Wellness.

[2] Turkmen N., Akal C., Özer B. (2019). Probiotic Dairy-Based Beverages: A Review. J. Funct. Foods.

[3] Arslan S. (2015). A Review: Chemical, Microbiological and Nutritional Characteristics of Kefir. CyTA—J. Food.

[4] Azizi N.F., Kumar M.R., Yeap S.K., Abdullah J.O., Khalid M., Omar A.R., Mohd A.O., Mortadza S.A.S., Alitheen N.B. (2021). Kefir and Its Biological Activities. Foods.

[5] Guzel-Seydim Z.B., Gökırmaklı Ç., Greene A.K. (2021). A Comparison of Milk Kefir and Water Kefir: Physical, Chemical, Microbiological and Functional Properties. Trends Food Sci. Technol..

[6] Chen T.-H., Wang S.-Y., Chen K.-N., Liu J.-R., Chen M.-J. (2009). Microbiological and Chemical Properties of Kefir Manufactured by Entrapped Microorganisms Isolated from Kefir Grains. J. Dairy Sci..

[7] Selhub E.M., Logan A.C., Bested A.C. (2014). Fermented Foods, Microbiota, and Mental Health: Ancient Practice Meets Nutritional Psychiatry. J. Physiol. Anthropol..

[8] Samardzic J., Jadzic D., Hencic B., Jancic J., Strac D.S., Samardzic J. (2018). Introductory Chapter: GABA/Glutamate Balance: A Key for Normal Brain Functioning. GABA and Glutamate—New Developments in Neurotransmission Research.

[9] Del Toro-Barbosa M., Hurtado-Romero A., Garcia-Amezquita L.E., García-Cayuela T. (2020). Psychobiotics: Mechanisms of Action, Evaluation Methods and Effectiveness in Applications with Food Products. Nutrients.

[10] Diez-Gutiérrez L., San Vicente L., Barrón L.J.R., del Carmen Villarán M., Chávarri M. (2020). Gamma-Aminobutyric Acid and Probiotics: Multiple Health Benefits and Their Future in the Global Functional Food and Nutraceuticals Market. J. Funct. Foods.

[11] Papadimitriou K., Zoumpopoulou G., Foligné B., Alexandraki V., Kazou M., Pot B., Tsakalidou E. (2015). Discovering Probiotic Microorganisms: In Vitro, in Vivo, Genetic and Omics Approaches. Front. Microbiol..

[12] Huebner J., Wehling R.L., Hutkins R.W. (2007). Functional Activity of Commercial Prebiotics. Int. Dairy J..

[13] Plessas S., Nouska C., Mantzourani I., Kourkoutas Y., Alexopoulos A., Bezirtzoglou E. (2016). Microbiological Exploration of Different Types of Kefir Grains. Fermentation.

[14] Chen Y.P., Hsiao P.J., Hong W.S., Dai T.Y., Chen M.J. (2012). Lactobacillus Kefiranofaciens M1 Isolated from Milk Kefir Grains Ameliorates Experimental Colitis in Vitro and in Vivo. J. Dairy Sci..

[15] Diosma G., Romanin D.E., Rey-Burusco M.F., Londero A., Garrote G.L. (2014). Yeasts from Kefir Grains: Isolation, Identification, and Probiotic Characterization. World J. Microbiol. Biotechnol..

[16] Zanirati D.F., Abatemarco M., de Cicco Sandes S.H., Nicoli J.R., Nunes Á.C., Neumann E. (2015). Selection of Lactic Acid Bacteria from Brazilian Kefir Grains for Potential Use as Starter or Probiotic Cultures. Anaerobe.

[17] García-Cayuela T., Korany A.M., Bustos I., de Cadiñanos L.P.G., Requena T., Peláez C., Martínez-Cuesta M.C. (2014). Adhesion Abilities of Dairy Lactobacillus Plantarum Strains Showing an Aggregation Phenotype. Food Res. Int..

[18] Brodkorb A., Egger L., Alminger M., Alvito P., Assunção R., Ballance S., Bohn T., Bourlieu-Lacanal C., Boutrou R., Carrière F. (2019). INFOGEST Static in Vitro Simulation of Gastrointestinal Food Digestion. Nat. Protoc..

[19] Tsukatani T., Higuchi T., Matsumoto K. (2005). Enzyme-Based Microtiter Plate Assay for γ-Aminobutyric Acid: Application to the Screening of γ-Aminobutyric Acid-Producing Lactic Acid Bacteria. Anal. Chim. Acta.

[20] Valenzuela J.A., Flórez A.B., Vázquez L., Vasek O.M., Mayo B. (2019). Production of γ-Aminobutyric Acid (GABA) by Lactic Acid Bacteria Strains Isolated from Traditional, Starter-Free Dairy Products Made of Raw Milk. Benef. Microbes.

[21] Balakrishnan N., Colton T., Everitt B., Piegorsch W., Ruggeri F., Balakrishnan N., Colton T., Everitt B., Piegorsch W., Ruggeri F., Teugels J.L. (2014). Wiley StatsRef: Statistics Reference Online.

[22] Gradilla-Hernández M.S., de Anda J., Garcia-Gonzalez A., Meza-Rodríguez D., Yebra Montes C., Perfecto-Avalos Y. (2020). Multivariate Water Quality Analysis of Lake Cajititlán, Mexico. Environ. Monit. Assess..

[23] Balouiri M., Sadiki M., Ibnsouda S.K. (2016). Methods for in Vitro Evaluating Antimicrobial Activity: A Review. J. Pharm. Anal..

[24] Everitt B., Landau S., Leese M., Stahl D., Everitt B. (2011). Cluster Analysis.

[25] Kim E., Cho E.-J., Yang S.-M., Kim M.-J., Kim H.-Y. (2021). Novel Approaches for the Identification of Microbial Communities in Kimchi: MALDI-TOF MS Analysis and High-Throughput Sequencing. Food Microbiol..

[26] Gantzias C., Lappa I.K., Aerts M., Georgalaki M., Manolopoulou E., Papadimitriou K., De Brandt E., Tsakalidou E., Vandamme P. (2020). MALDI-TOF MS Profiling of Non-Starter Lactic Acid Bacteria from Artisanal Cheeses of the Greek Island of Naxos. Int. J. Food Microbiol..

[27] Krausova G., Hyrslova I., Hynstova I. (2019). In Vitro Evaluation of Adhesion Capacity, Hydrophobicity, and Auto-Aggregation of Newly Isolated Potential Probiotic Strains. Fermentation.

[28] Dlamini Z.C., Langa R.L.S., Aiyegoro O.A., Okoh A.I. (2019). Safety Evaluation and Colonisation Abilities of Four Lactic Acid Bacteria as Future Probiotics. Probiotics Antimicrob. Proteins.

[29] Gad G.F.M., Abdel-Hamid A.M., Farag Z.S.H. (2014). Antibiotic Resistance in Lactic Acid Bacteria Isolated from Some Pharmaceutical and Dairy Products. Braz. J. Microbiol..

[30] Huddleston J.R. (2014). Horizontal Gene Transfer in the Human Gastrointestinal Tract: Potential Spread of Antibiotic Resistance Genes. Infect. Drug Resist..

[31] Lerner A., Matthias T., Aminov R. (2017). Potential Effects of Horizontal Gene Exchange in the Human Gut. Front. Immunol..

[32] Obioha P.I., Ouoba L.I.I., Anyogu A., Awamaria B., Atchia S., Ojimelukwe P.C., Sutherland J.P., Ghoddusi H.B. (2021). Identification and Characterisation of the Lactic Acid Bacteria Associated with the Traditional Fermentation of Dairy Fermented Product. Braz. J. Microbiol..

[33] Hu C., Ren L., Zhou Y., Ye B. (2019). Characterization of Antimicrobial Activity of Three *Lactobacillus Plantarum* Strains Isolated from Chinese Traditional Dairy Food. Food Sci. Nutr..

[34] Arena M.P., Silvain A., Normanno G., Grieco F., Drider D., Spano G., Fiocco D. (2016). Use of Lactobacillus Plantarum Strains as a Bio-Control Strategy against Food-Borne Pathogenic Microorganisms. Front. Microbiol..

[35] Talib N., Mohamad N.E., Yeap S.K., Hussin Y., Aziz M.N.M., Masarudin M.J., Sharifuddin S.A., Hui Y.W., Ho C.L., Alitheen N.B. (2019). Isolation and Characterization of Lactobacillus Spp. from Kefir Samples in Malaysia. Molecules.

[36] Escobar-Ramírez M.C., Jaimez-Ordaz J., Escorza-Iglesias V.A., Rodríguez-Serrano G.M., Contreras-López E., Ramírez-Godínez J., Castañeda-Ovando A., Morales-Estrada A.I., Felix-Reyes N., González-Olivares L.G. (2020). Lactobacillus Pentosus ABHEAU-05: An in Vitro Digestion Resistant Lactic Acid Bacterium Isolated from a Traditional Fermented Mexican Beverage. Rev. Argent. Microbiol..

[37] Hurtado-Romero A., Del Toro-Barbosa M., Garcia-Amezquita L.E., García-Cayuela T. (2020). Innovative Technologies for the Production of Food Ingredients with Prebiotic Potential: Modifications, Applications, and Validation Methods. Trends Food Sci. Technol..

[38] Chatterjee E., GA Manuel S. (2016). Effect of Fruit Pectin on Growth of Lactic Acid Bacteria. J. Probiotics Health.

[39] Figueroa-GonzáLez I., RodríGuez-Serrano G., GóMez-Ruiz L., GarcíA-Garibay M., Cruz-Guerrero A. (2019). Prebiotic Effect of Commercial Saccharides on Probiotic Bacteria Isolated from Commercial Products. Food Sci. Technol..

[40] De Souza Oliveira R.P., Rodrigues Florence A.C., Perego P., De Oliveira M.N., Converti A. (2011). Use of Lactulose as Prebiotic and Its Influence on the Growth, Acidification Profile and Viable Counts of Different Probiotics in Fermented Skim Milk. Int. J. Food Microbiol..

[41] Ayala Monter M.A., Hernández Sánchez D., Pinto Ruiz R., González Muñoz S.S., Bárcena Gama J.R., Hernández Mendo O., Torres Salado N. (2018). Efecto Prebiótico de Dos Fuentes de Inulina En El Crecimiento in Vitro de Lactobacillus Salivarius y Enterococcus Faecium. Rev. Mex. Cienc. Pecu..

[42] Trapala J., Bustos-Jaimes I., Manzanares P., Bárzana E., Montiel C. (2020). Purification and Characterization of an Inulinase Produced by a Kluyveromyces Marxianus Strain Isolated from Blue Agave Bagasse. Protein Expr. Purif..

[43] García Gamboa R., Ortiz Basurto R.I., Calderón Santoyo M., Bravo Madrigal J., Ruiz Álvarez B.E., González Avila M. (2018). In Vitro Evaluation of Prebiotic Activity, Pathogen Inhibition and Enzymatic Metabolism of Intestinal Bacteria in the Presence of Fructans Extracted from Agave: A Comparison Based on Polymerization Degree. LWT.

[44] Redruello B., Saidi Y., Sampedro L., Ladero V., del Rio B., Alvarez M.A. (2021). GABA-Producing Lactococcus Lactis Strains Isolated from Camel’s Milk as Starters for the Production of GABA-Enriched Cheese. Foods.

[45] Bhanwar S., Bamnia M., Ghosh M., Ganguli A. (2013). Use of *Lactococcus Lactis* to Enrich Sourdough Bread with γ-Aminobutyric Acid. Int. J. Food Sci. Nutr..

[46] Perpetuini G., Tittarelli F., Battistelli N., Suzzi G., Tofalo R. (2020). Γ-aminobutyric Acid Production by *Kluyveromyces Marxianus* Strains. J. Appl. Microbiol..

[47] Demirbaş F., İspirli H., Kurnaz A.A., Yilmaz M.T., Dertli E. (2017). Antimicrobial and Functional Properties of Lactic Acid Bacteria Isolated from Sourdoughs. LWT—Food Sci. Technol..

[48] Gómez de Cadiñanos L.P., García-Cayuela T., Martínez-Cuesta M.C., Peláez C., Requena T. (2019). Expression of Amino Acid Converting Enzymes and Production of Volatile Compounds by Lactococcus Lactis IFPL953. Int. Dairy J..

[49] Öztürkoğlu Budak S., Akal H.C., Öztürkoğlu Budak Ş., Akal H.C. (2018). Microbial Cultures and Enzymes in Dairy Technology.

